# Exposure to nicotine-derived nitrosamine ketone and arecoline synergistically facilitates tumor aggressiveness via overexpression of epidermal growth factor receptor and its downstream signaling in head and neck squamous cell carcinoma

**DOI:** 10.1371/journal.pone.0201267

**Published:** 2018-08-27

**Authors:** Shih-Hsien Yang, Tsai-Yu Lee, Chun An Ho, Chin-Yuh Yang, Wen-Yen Huang, Yu-Chun Lin, Shin Nieh, Yaoh-Shiang Lin, Su-Feng Chen, Fu-Huang Lin

**Affiliations:** 1 Graduate Institute of Medical Sciences, National Defense Medical Center, Taipei, Taiwan; 2 Department of Medical Administration Office, National Defense Medical Center & Tri-Service General Hospital Beitou Branch, Taipei, Taiwan; 3 Tri-Service General Hospital Songshan Branch, National Defense Medical Center, Division of Colon and Rectum Surgery, Department of Surgery, Taipei, Taiwan; 4 Tri-Service General Hospital, National Defense Medical Center, Division of Colon and Rectum Surgery, Department of Surgery, Taipei, Taiwan; 5 Department of Pathology, National Defense Medical Center & Tri-Service General Hospital, Taipei, Taiwan; 6 Department of Dentistry, Cheng Hsin Hospital, Taipei, Taiwan; 7 Department of Radiation Oncology, Tri-Service General Hospital, National Defense Medical Center, Taipei, Taiwan; 8 Institute of Clinical Medicine, National Yang-Ming University, Taipei, Taiwan; 9 Department of Otorhinolaryngology, Head and Neck Surgery, Kaohsiung Veterans General Hospital, Kaohsiung, Taiwan; 10 Department of Dental Hygiene and School of Dentistry, China Medical University, Taichung, Taiwan; 11 School of Public Health, National Defense Medical Center, Taipei, Taiwan; Taipei Medical University College of Medicine, TAIWAN

## Abstract

Long-term nicotine-derived nitrosamine ketone (NNK) and arecoline exposure promotes carcinogenesis and head and neck squamous cell carcinoma (HNSCC) progression, although most associated data on the two were analyzed individually. The molecular mechanisms underlying tumor progression associated with the synergistic effects of NNK and arecoline remain unclear. We treated SCC-25 and FaDu cells with NNK and arecoline (separately or in combination) for 3 months. Comparative analysis was performed to investigate the mechanism underlying the acquisition of properties related to tumor promotion, including stemness, anti-apoptosis, and resistance to HNSCC therapeutics. Long-term exposure to NNK and arecoline resulted in an increase in cancer stem cell properties, anti-apoptosis, and the resistance to cisplatin in HNSCC. We detected abundant epidermal growth factor receptor (EGFR) expression in HNSCC cells after combined treatment with NNK and arecoline. EGFR was pivotal in inducing tumor promotion and anti-apoptosis in cancer cells by inducing pAKT and NFκB. Combined treatment with NNK and arecoline synergistically facilitated tumor aggressiveness via EGFR–AKT signaling. Targeting EGFR–AKT signaling may be a feasible strategy for treating HNSCC.

## Introduction

Head and neck squamous cell carcinoma (HNSCC) is one of the ten most common cancers in Taiwan and worldwide.[1, 2] In general, HNSCC occurs in the oral cavity, oropharynx, hypopharynx, larynx, and paranasal sinuses. Due to the complicated anatomy of the head and neck, head and neck cancer involves one of the most difficult surgical treatments; therefore, multidisciplinary and diverse treatment strategies are needed. Despite advances and improvements in diagnostic and surgical techniques, chemotherapy, and radiotherapy, the prognosis of patients with HNSCC remains unchanged.[3, 4] Metastases and treatment failures are thought to be responsible for most deaths associated with HNSCC. Understanding the mechanisms underlying tumorigenesis, metastases, and treatment failure may help reduce the morbidity and mortality of HNSCC. Thus, a better understanding of the molecular mechanism of HNSCC aggressiveness is urgently needed to promote the development of a more efficient therapeutic target and to identify key pathways mediating disease progression.

The tobacco-related carcinogen nitrosamine 4-(methylnitrosamino)-1-(3-pyridyl)-1-butanone, also known as nicotine-derived nitrosamine ketone (NNK), is a major hazard component in cigarette content and has been recognized as its most potent carcinogen.[5, 6] Tobacco smoking with long-term exposure to NNK, as well as heavy arecoline consumption due to habitual betel nut chewing, have been associated with increased risks for tumorigenesis of head and neck cancers, including in the oral cavity, pharynx, larynx, and esophagus. It also appears that smoking and betel nut chewing are the two most common distinguishing risk factors for HNSCC progression and play pivotal roles in increasing cancer cell growth and survival. Arecoline is a predominant psychoactive agent in areca nuts.[7] Some effects of the areca nut are euphoric or anxiolytic, as with NNK.[8, 9] Based on a large-scale analysis of smoking or consumption of betel quid versus the incidence of HNSCC,[10, 11] arecoline and NNK are thought to be associated with poor responses to chemoradiotherapy and shorter overall survival in patients. Nicotine in tobacco is modified by nitrosation to form nitrosamines and the well-known tobacco-specific carcinogen, NNK, which was reported to enhance cancer progression and metastasis through α7-nAChR and to be a hallmark of the epithelial–mesenchymal transition (EMT).[12] NNK binds the β-adrenoceptor (β-AR) and induces cell proliferation and invasion in pancreatic cancer. The β2-adrenergic antagonist was shown to reduce the activation of NF-κB, extracellular signal-regulated kinase, and Akt-related pathways, resulting in cell death.[13, 14] Arecoline exhibits similar carcinogenic and long-term toxic effects as NNK, and both molecules are alkaloids with comparable structures. Arecoline is a full agonist of acetylcholine muscarinic receptors, and its activity is probably mediated by muscarinic M3 receptors found in the smooth muscles of the blood vessels. Areca-nut chewing was popular in many parts of Asia to induce salivation and euphoria.[15] Activation of muscarinic receptors can lead to Akt stimulation, which inhibits apoptosis and promotes cell survival. The expression of several proteins with aberrant regulation has been found in association with oral cancer, including the epidermal growth factor receptor (EGFR), Akt, and GSK3β.[16–18] Chronic exposure to arecoline promotes the acquisition of cancer stemness, EMT, and chemo-resistance.[19, 20]

Cancer stem cells (CSCs) have been identified in many solid tumors, including breast, prostate, and pancreatic carcinomas.[21] CSCs show a high capability for tumor initiation, motility, and invasion, with the overexpression of representative markers such as CD24 and CD44 and the activity of aldehyde dehydrogenase 1 (ALDH-1) being associated with stem cell-like properties.[21–24] Persistent cytotoxicity promotes the activation of CSCs, resulting in treatment failure and relapse, and the use of these substances has been previously associated with cancer incidence and cancer progression.[21] The exact mechanism(s) and cross-linked effects of NNK and arecoline underlying tumor progression in HNSCC remain unclear.

As we reported previously,[25] long-term NNK exposure increases anti-apoptosis and therapeutic resistance via the Snail-RKIP signaling pathway. Here, we utilized our non-adhesive culture system to investigate the characteristics of HNSCC cells following long-term and combined treatment with NNK and arecoline. The aim of this study, therefore, was to validate the effects of two major risk factors and the associated signaling pathway involved in modulating tumor growth, apoptosis, and stem cell properties. Our current findings provide insight into the molecular mechanism underlying HNSCC and reveal a possible therapeutic strategy for improving HNSCC prognosis.

## Materials and methods

### Preparation of cells and subsequent sphere culture

Six HNSCC cell lines were initially prepared and tested and details were seen in our previous report.[25] Two representative HNSCC cell lines, SCC25 (catalog number CRL-1628) and FaDu (catalog number HTB-43), were chosen for subsequent study The cells were cultured in 10-cm dishes with a non-adhesive surface. The 10-cm dishes were made to be non-adhesive by coating them with a thin agarose film. Cells were plated and the culture medium was changed every other day until sphere formation occurred, as we described previously.[26]

### Long-term exposure of cells to NNK and arecoline

NNK was obtained from ChemSyn Laboratories (Lenexa, KS). Arecoline was purchased from Sigma Chemical Co. (St. Louis, MO, USA) and dissolved in DMSO to a concentration of 100 mM. Parental SCC25 and FaDu cells were plated at a density of 5 × 10^4^ live cells/10-cm dish and treated with arecoline at a final concentration of 0, 10, 50, 100, or 150 nM for 3 months. Then the cells were harvested and cultured with NNK (50 nM) and/or arecoline (130 nM), with the medium being changed every other day. Arecoline (130 nM) and NNK (50 nM) were tested and found to be the most optimal dose for further analysis. Three experimental groups were prepared to examine the effects of NNK and arecoline, alone or in combination. The first and second groups of HNSCC cells were treated with NNK and arecoline separately, and the third group was treated with both NNK and arecoline.

### Cell viability assay

The cells were seeded in a 6-well plate at a density of 3 × 10^4^ cells/well, and cell proliferation was measured by the 3-(4,5-dimethyl-thiazol-2-yl)-2,5-diphenyltetrazolium bromide (MTT) reduction method. After incubation with NNK and arecoline at different concentrations for 0, 24, 48, or 72 h or more than 3 months, the cells were incubated with 2.5% MTT solution (5 mg/ml) for cell viability assay, as previously reported. [25] The same test was repeated three times, and the optical density was calculated for statistical analysis.

### Apoptosis assay

Apoptotic cells were detected based on annexin V expression (Gene Research, Taipei, Taiwan) according to the manufacturer’s guidelines. After staining, the cells were incubated for 30 min in the dark at room temperature with 5 μl of a fluorescein isothiocyanate (FITC)-conjugated anti-annexin V antibody. The cells were then analyzed with a FACSCalibur instrument (Becton Dickinson, San Diego, CA, USA).

### Migration assay

A total of 1 × 10^5^ cells was seeded in the top chamber of a 24-well plate with micropore polycarbonate membrane filters containing 8-μm pores (Becton Dickinson Labware, Lincoln Park, NJ). The bottom chamber was filled with RPMI 1640 medium containing 10% fetal bovine serum as a chemoattractant. The migrated cells were harvested after 24 h and stained with hematoxylin. The migrated cancer cells were then visualized and counted from 5 different visual fields (magnification, 100×) under a light microscope.

### Invasion assay

The 24-well plate Transwell^®^ system with a polycarbonate membrane filter was employed to evaluate the invasion ability of cells and the details were followed by previous protocol. [25]

### Western blot analysis

Whole cell lysates (50 μg) were separated by electrophoresis on 12.5% denaturing polyacrylamide gels. The membranes were incubated overnight at 4°C with primary antibodies (0.1 μg/ml) against Oct4, Nanog, β-AR and phospho EGFR were purchased from Abcam Corporation (Abcam, Cambridge, UK), Snail, Twist, Fibronectin, E-cadherin, CD21, CD44, CD133, ALDH-1, phospho AKT, phospho NFκB, α7-nAChR, Bcl-2 were acquired from Santa Cruz Biotechnology (Santa Cruz, CA). Bax was obtained from Becton, Dickinson and Company (BD, Transduction Laboratories TM), MDR-1 and ABCG2 were purchased from Millipore Corporation (Millipore Corporation, Billerica, MA, USA), caspase-3 and PARP were obtained from Invitrogen Corporation (Invitrogen, Camarillo, CA, USA) in Tris-Tween-Buffer-Saline buffer containing 3% non-fat milk. Subsequently, each membrane was washed and incubated for 1 h at 25°C with a secondary anti-mouse, anti-rabbit, or anti-goat antibody conjugated with horseradish peroxidase (1: 1000; Santa Cruz Biotechnology, Inc.), as previously reported. [25]

### Chemosensitivity assay

Cells were seeded in 10-cm dishes at a density of 1 × 10^6^ cells/dish. For the chemosensitivity assay, cells were treated with 0 to 100 μM cisplatin (Cis; Sigma–Aldrich) for 48 h. Relative cell survival was determined by the MTS assay using the CellTiter 96 Aqueous One Solution Cell Proliferation Assay Kit (Promega, Madison, WI, USA).

### Flow cytometry

A single-cell suspension of 1 × 10^6^ cells containing trypsinized cells and spheres was suspended in 1 mL of phosphate-buffered saline (PBS) and ALDH1 (ALDEFLUOR Assay Kit; Stem Cell Technologies, Durham, NC) as previously reported. [25]

### Statistical analysis

All data are shown as the mean ± SD. Differences between groups were calculated using Student’s *t*-test. Dose-dependent effects were measured by linear regression. All statistical analyses were performed using SPSS software, version 15.0 (SPSS, Inc., Chicago, IL, USA).

## Results

### Long-term exposure to NNK and arecoline promoted migration and invasion and enhanced the EMT with morphological alterations in HNSCC cells

**Fig 1:**
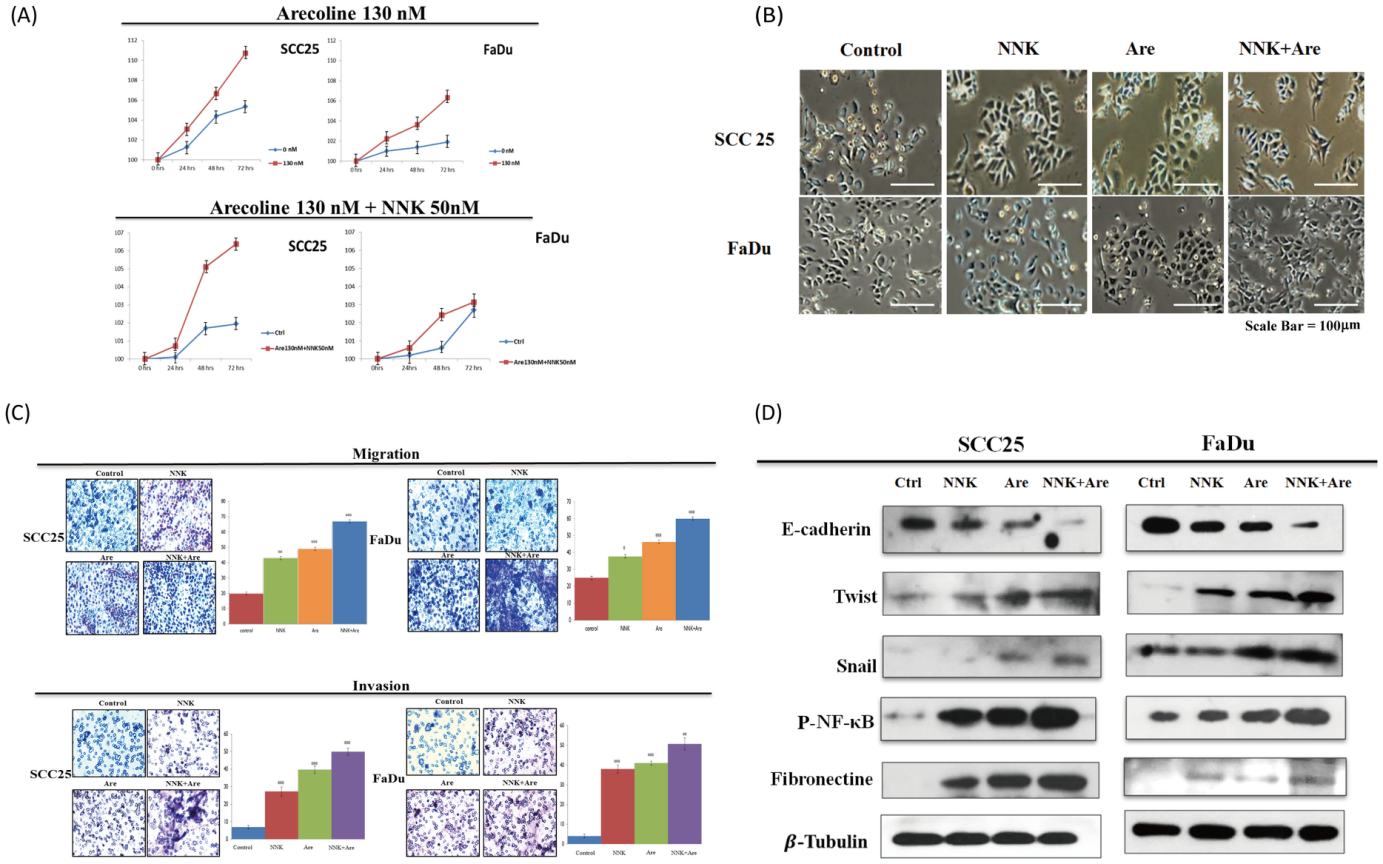
Promotion of proliferation, enhancement of migration and invasion characteristics of the EMT phenomenon, and morphological alterations following long-term exposure to NNK and arecoline. **A**. Proliferation assays showed that treatment with NNK or arecoline increased the growth rate in a short-term dependent manner in both SCC25 and FaDu cells; however, long-term treatment with NNK and arecoline caused sluggish cell proliferation until growth equilibrium was reached. **B**. As indicated by the arrows, long-term treatment with NNK, arecoline, or both NNK and arecoline induced EMT with a characteristic cell morphology observed for both cell lines. **C**. Long-term exposure to NNK, arecoline, or the combination of NNK and arecoline significantly increased the migration and invasion of HNSCC cells (*p* < 0.05). **D**. Regarding the EMT-like properties in HNSCC cells, western blot analysis revealed that long-term exposure to NNK, arecoline, and both in combination also induced alterations in the expression of representative EMT markers showing decreased expression of E-cadherin, increased expression of fibronectin, and increased expression of EMT regulators (Snail, Twist, and NFκB) in both cell lines. A synergistic effect was found in the group treated with NNK and arecoline in combination, showing more sensitivity than the other two experimental groups (*p* < 0.05).

We first determined the effects of NNK and arecoline exposure on cell propagation in three experimental groups of HNSCC cells by performing proliferation assays. Following a 3-month exposure to various concentrations of arecoline, SCC25 and FaDu cells were stimulated with NNK (50 nM), arecoline (130 nM), or a combination of NNK (50 nM) and arecoline (130 nM) for 24 h, 48 h, 72 h, or 3 months, as previously reported. [25] Proliferation assay results showed that NNK and arecoline enhanced the cell growth rate in the short term in both cell lines; however, long-term treatment with NNK and arecoline eventually slowed cell proliferation until growth equilibrium was achieved. This standard condition was used for further experiments (Fig 1A).

Next, we investigated differences in the effects of NNK, arecoline, and combined treatment on migration and invasion and whether the treatments were involved in the EMT of HNSCC cells. EMT occurs during embryogenesis and is also a sign of tumor cell instability, resulting in the migration to neighborhood tissues. Long-term alkaloid exposure induced EMT in both cell lines, with a morphological conversion from an epithelioid to a mesenchymal appearance (Fig 1B). To determine the effect of alkaloids on invasion ability, HNSCC cells were incubated with 50 nM NNK, 130 nM arecoline, or a combination of 50 nM NNK and 130 nM arecoline, after which they were prepared for invasion and migration assays. Combination treatment with NNK and arecoline promoted greater degrees of invasion and migration than treatment with either reagent alone (*p* < 0.05) (Fig 1C). Western blot analysis revealed that long-term exposure to NNK, arecoline, and their combination altered the expression of representative EMT markers, including decreased E-cadherin expression, increased fibronectin expression, and increased expression of EMT regulators (Snail, Twist, and NFκB) in both cell lines. A synergistic effect of combination treatment was found, based on the overexpression of all of the aforementioned markers, especially NFκB (Fig 1D).

### Long-term NNK and arecoline exposure promoted sphere formation and the overexpression of stem cell markers in HNSCC cells

**Fig 2:**
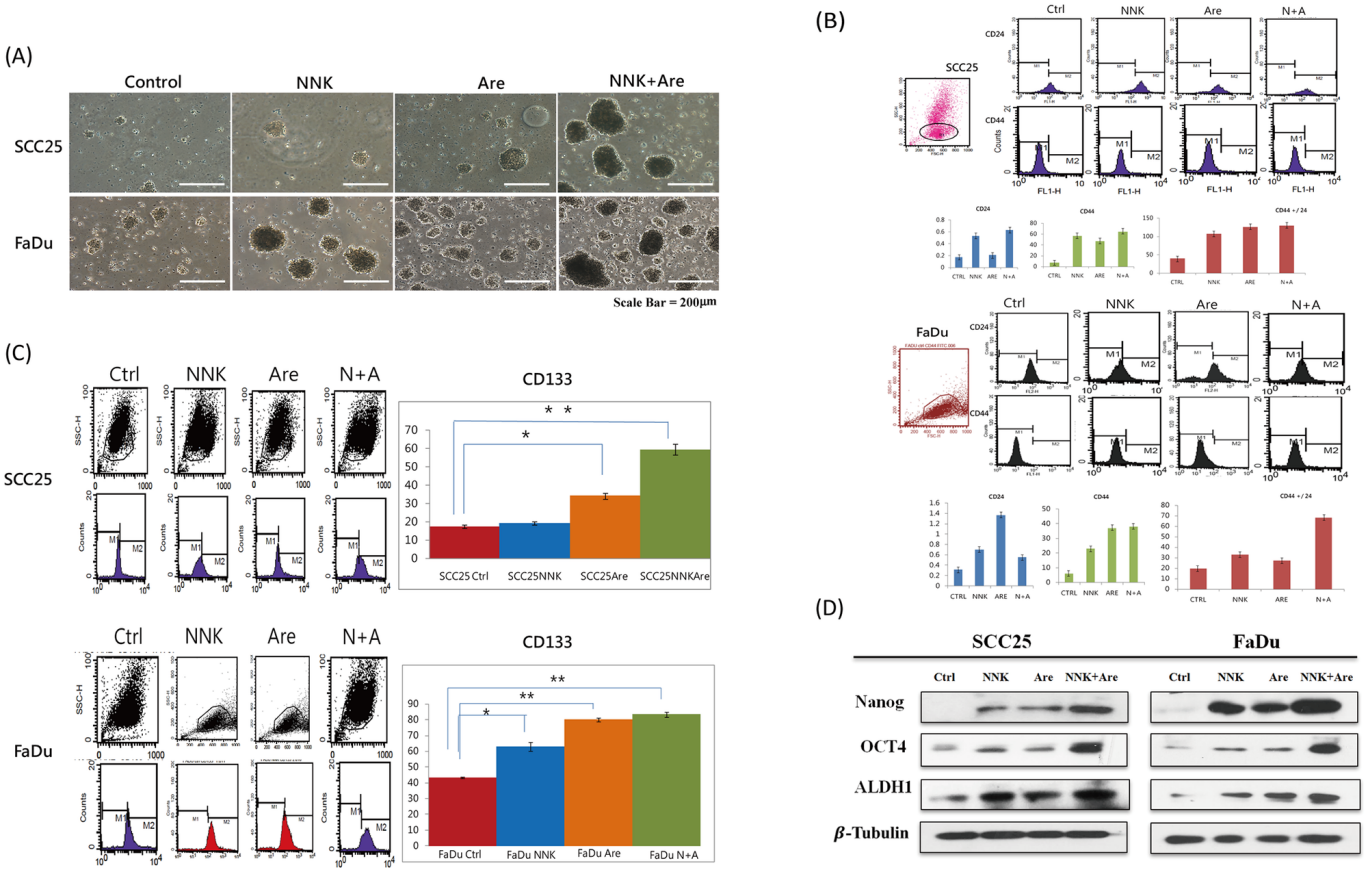
Characterization of the sphere-forming ability and expression of CSC markers following long-term exposure to NNK and arecoline. **A**. Morphological transformations under each condition through the stimulation of sphere formation by long-term alkaloid treatment were observed using a non-adhesive culture system.[26] **B. C**. Flow cytometric analysis showed significant overexpression (*p* < 0.05) of CSC representative markers, including CD24, CD44, and CD133. **D**. Western blot analysis also showed upregulation of Nanog, OCT4, and ALDH-1 in the three experimental groups compared with that in control cells. A prominent synergistic effect was observed in the group treated with NNK and arecoline in combination in terms of the upregulation of representative CSC markers.

The efficiency of sphere formation was evaluated to examine self-renewal in CSCs following long-term NNK, arecoline, and combined exposure using a non-adhesive culture system. Most cell clusters transformed into spheres after 7 d in culture. In contrast, control cells only formed irregular cell masses that lacked a spheroid appearance (Fig 2A). Flow cytometric analysis of the representative CD133 and CD24/CD44^+^ markers showed significant overexpression in long-term alkaloid-treated cells compared with that in parental cells (*p* < 0.05) (Fig 2B and 2C). Identification of common CSC markers for HNSCC also showed upregulation of Nanog, OCT-4, and ALDH-1 in cells exposed to long-term alkaloid treatment compared with that in the parental cell lines (Fig 2D). Again, a synergistic effect involving the overexpression of all aforementioned CSC markers was found in the experimental group exposed to combination treatment.

### Long-term exposure to NNK and arecoline increased drug resistance and upregulated MDR1 and ABCG2 expression

**Fig 3:**
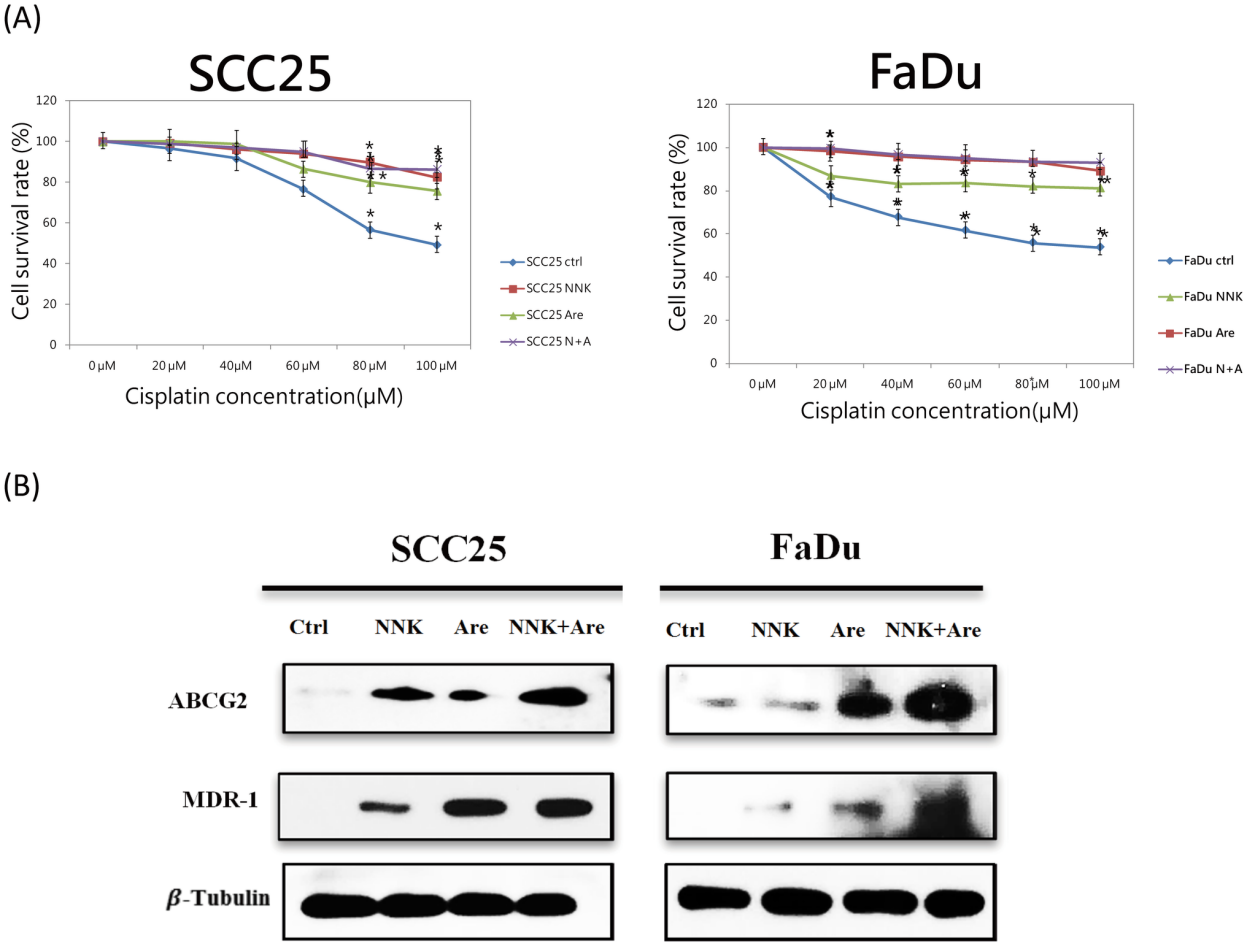
Comparison of drug resistance and drug resistance-related gene products following exposure to NNK and arecoline. **A**. Drug sensitivity after treatment with different doses of Cis for 24 h revealed marked differences between long-term alkaloid-treated cells and parental cells. Drug resistance was higher in long-term alkaloid-treated cells than in the parental cells (*p* < 0.05). **B**. Western blot analysis showed upregulation of the drug-resistance-related proteins ABCG2 and MDR-1. Alkaloid treatment upregulated both the *MDR1* and *ABCG2* gene products, suggesting that long-term exposure to NNK and arecoline caused greater resistance to Cis than that in untreated, parental cells. A synergistic effect was also found in the group exposed to combination treatment with NNK and arecoline, as this group exhibited more sensitivity than the other two experimental groups (*p* < 0.05).

The observation that alkaloids enhanced the CSC population suggested their involvement in modulating chemoresistance, an important characteristic of CSCs.[27] To investigate the expression profile of alkaloid-exposed cells and chemoresistance, control and alkaloid-exposed cells were treated with Cis and subjected to MTT assays and western blot analysis. Significant differences in chemosensitivity were found between long-term alkaloid-treated cells and parental cells after exposure to various Cis concentrations for 24 h (*p* < 0.05) (Fig 3A). Western blotting revealed that long-term alkaloid exposure resulted in overexpression of the *MDR1* and *ABCG2* genes compared to that in the parental cells, which was enhanced synergistically in cells treated with both NNK and arecoline (Fig 3B).

### Long-term exposure to NNK and arecoline enhanced anti-apoptosis signaling

**Fig 4:**
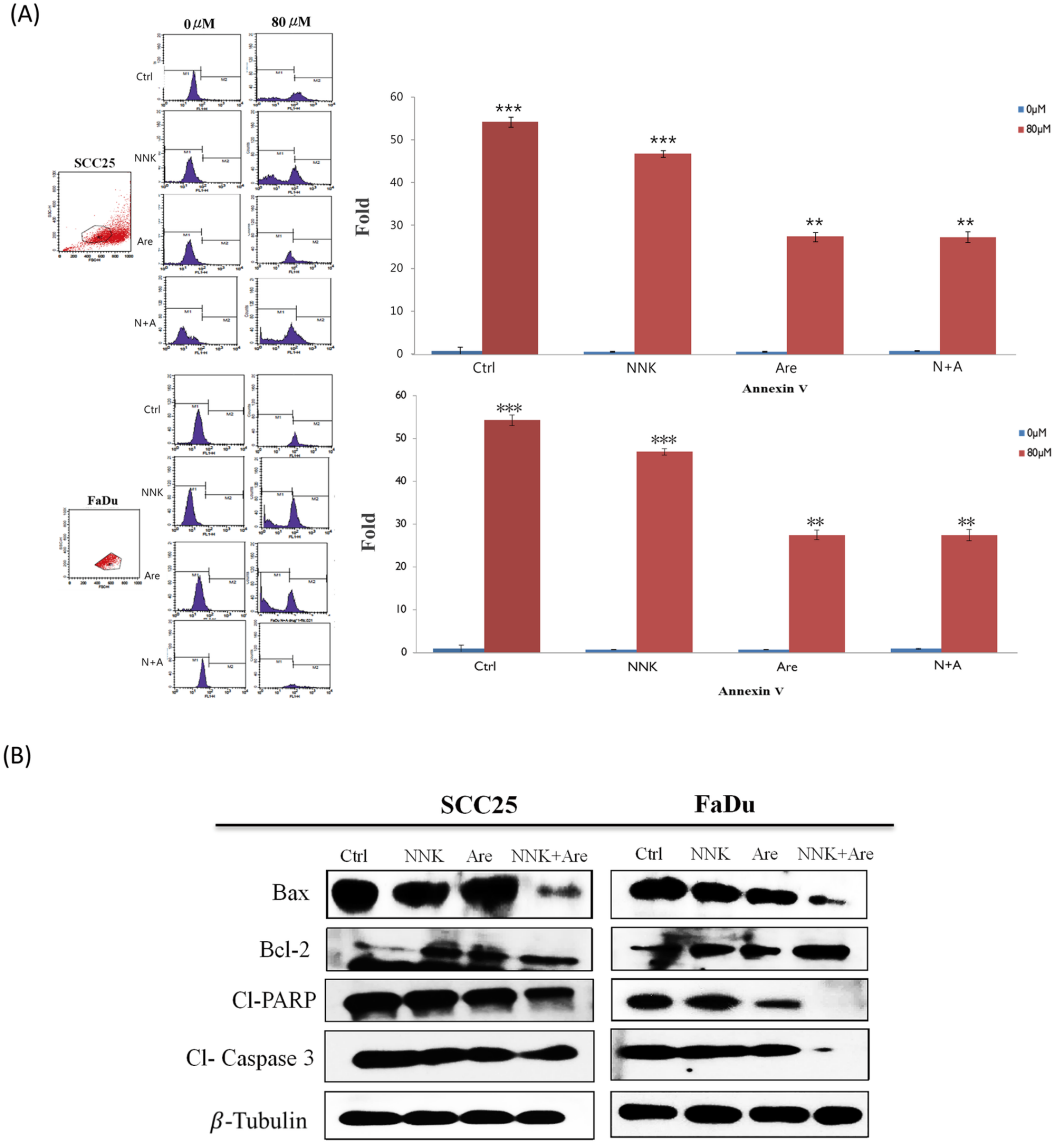
Characterization of the anti-apoptotic ability following long-term exposure to NNK and arecoline. **A**. Apoptosis analysis via annexin V staining showed that long-term alkaloid treatment attenuated apoptotic activity in both cell lines (*p* < 0.05). **B**. Western blot analysis demonstrated that treated cells displayed anti-apoptotic properties, such as increased Bcl-2 expression and downregulation of apoptotic-related proteins, including Bax, Cl-caspase-3, and Cl-PARP.

Flow cytometric analysis (using annexin V staining) showed that long-term exposure to NNK and arecoline significantly attenuated (*p* < 0.05) apoptosis in both cell lines following Cis treatment (80 μM), as shown in Fig 4A. Immunoblotting demonstrated that long-term NNK- and arecoline-treated cells showed dramatically increased levels of the anti-apoptotic oncoprotein Bcl-2 in both cell lines. In contrast, the levels of the apoptosis-promoting proteins Bax, cleaved caspase-3 (cl-caspase 3), and cleaved-poly (ADP-ribose) polymerase (Cl-PARP) decreased in both HNSCC cell lines exposed to long-term NNK and arecoline treatment (Fig 4B). Again, a synergistic alteration in the expression of apoptotic markers was found in the combination treatment experimental group.

### Long-term exposure to NNK and arecoline induced the activation of EGFR but not the α7 nicotinic acetylcholine receptor (α7-nAChR) and β-AR

**Fig 5:**
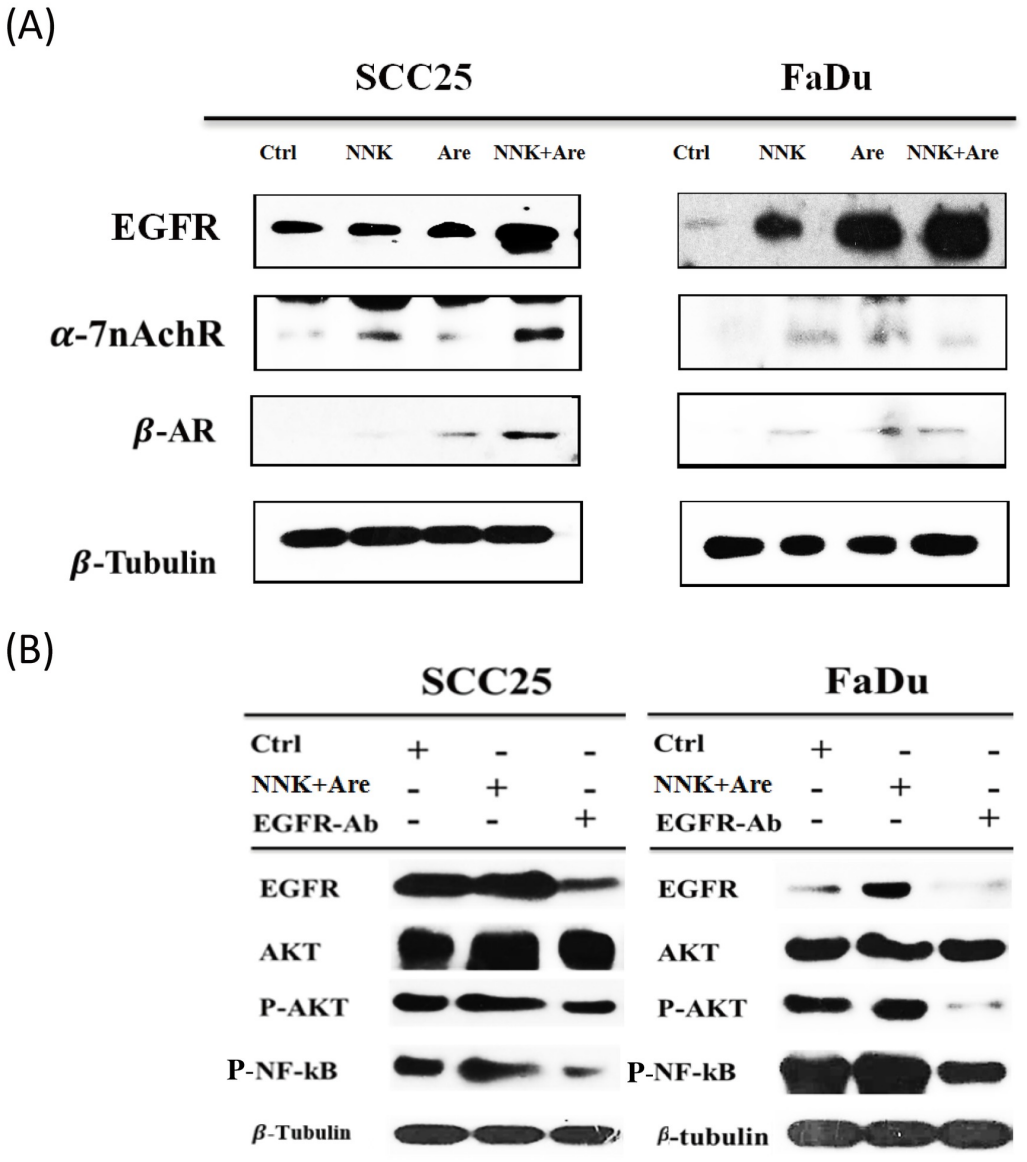
Induction and activation of EGFR effector molecules (other than α7-nAChR and β-AR) following long-term exposure to NNK and arecoline. EGFR was critical for the proliferation of HNSCC cells induced by long-term NNK and arecoline treatment. **A**. The alkaloid receptors EGFR, α7-nAChR, and β-AR were detected by western blotting. However, following long-term exposure to NNK and arecoline, HNSCC cells strongly expressed EGFR, but not α7-nAChR and β-AR. **B**. Treatment with an anti-EGFR antibody downregulated EGFR signaling after long-term NNK, arecoline, and combined NNK and arecoline treatments. Western blot analysis showed that NNK and arecoline induced p-AkT and NFκB in SCC25 and FaDu cells, which was diminished in the presence of the anti-EGFR antibody.

EGFR, α7-nAChR, and β-AR each play pivotal roles in regulating the alkaloid-induced growth and progression of cancer cells. Here, we determined whether alkaloids could synergistically affect the activation of EGFR following long-term exposure. Western blot analysis showed that α7-nAChR stimulation was mainly induced by NNK and that β-AR expression was slightly induced by arecoline. Nevertheless, in cells treated with a combination of NNK and arecoline, abundant EGFR expression was stimulated in a long-term manner (Fig 5A). In addition, treating cells with anti-EGFR antibody to neutralize EGFR activity resulted in downregulation of phospho-EGFR expression induction of the downstream phosphor-AKT and NFκB proteins caused by long-term treatment with NNK and arecoline in SCC25 and FaDu cells (Fig 5B). Collectively, these results support our hypothesis that EGFR plays a more critical role is a pivotal receptor in promoting the proliferation of HNSCC cells following long-term treatment with NNK and arecoline.

## Discussion

Long-term exposure to environmental chemicals such as NNK and arecoline can increase tumor initiation and the aggressiveness of existing tumors, especially in HNSCC. However, the underlying mechanisms and signaling pathways responsible for tumor progression following long-term exposure to NNK and arecoline in combination have not been studied and need further clarification. This study was designed to include experimental groups with long-term (3-month) NNK and/or arecoline exposure to mimic habitual smoking and/or betel-nut chewing in daily life, which represented a longer and higher exposure than those used in previously reported studies conducted over a 2-month period.[20, 25] Long-term exposure to NNK has been reported to be associated with tumor initiation and promotion. Previous data showed the importance of α7nAChR in NNK-induced cell proliferation for lung cancer, breast cancer, and pancreatic cancer.[28, 29] Previous reports also showed that arecoline exerted a partial agonist activity with α7-nAChR in a dose-dependent manner or with habitual use. [30, 31] Areca is of much more significance as a drug of abuse because it is a selective partial agonist of both α4- and α6-nAChR. The silent agonist activity of arecoline for α7-nAChR indicated a mechanism for its effects on immune cells and also revealed its significant potential involvement in the carcinogenic effects of areca use.

Accumulating evidence has also demonstrated that β-adrenergic receptors mediate the proliferative and anti-apoptotic effects of NNK in non-lung cancer cells.[32] Furthermore, the mitogenic effects of NNK have been found to be mediated by β-adrenergic receptors, indicating that NNK promotes the growth of gastric cancers via PKC and ERK1/2 phosphorylation in a β-adrenergic receptor-dependent fashion.[33, 34] Our previous results showed that NNK induces HNSCC cell proliferation through a α7-nAChR–EGFR signaling axis to facilitate the growth of HNSCC. Our results from this study demonstrated that the arecoline-induced proliferation of HNSCC cells was mediated by both EGF and β-adrenergic receptors. However, the effects on EGFR and AKT signaling have been reported to be involved in cancer cell growth, up-regulating downstream anti-apoptosis signaling, sphere-forming capability, invasive and migratory potentials, and chemoresistance in numerous cancers.[35–39] In this regard, our results clearly verified that dual activation of EGFR following long-term treatment with NNK and arecoline promoted cancer proliferation more robustly than NNK or arecoline treatment alone.

Following long-term exposure to NNK and arecoline, EGFR effector molecules (other than α7-nAChR and β-AR) were prominently induced and activated. Furthermore, we also demonstrated that abolishing EGFR activation by antibody treatment suppressed AKT and NFκB phosphorylation and cancer cell activation in HNSCC. EGFR overexpression in response to AKT phosphorylation and NFκB activation inhibited apoptosis, which has been correlated with low patient survival rates.[40] We successfully confirmed that pAKT and NFκB activity (via the EGFR–AKT signaling pathway) played significant roles in the proliferation of SCC25 and FaDu cells. Previous data have demonstrated that the suppression of apoptotic pathways played an important role in response to long-term stimulation with alkaloids.[41–45] Our data clearly demonstrated that downregulation of apoptotic proteins including CL-PARP and cl-caspase 3 supervened following long-term treatment with NNK and arecoline in both cell lines. The Bcl-2 family of proteins is well-characterized regulators of apoptosis. The pro-apoptotic protein Bax can act as a gateway for caspase-mediated cell death. The Bcl-2:Bax ratio is an important determinant of the susceptibility to apoptosis,[37, 46] which was also confirmed by our results.

The EMT is a critical step in the development of metastasis and acquisition of resistance to targeted therapeutics, including tyrosine kinase inhibitors against EGFR. Replacement of the epithelial marker E-cadherin with the mesenchymal marker fibronectin significantly was shown to increase Snail expression and slightly increase Twist expression, which together represent typical features of EMT. EGFR/AKT-mediated signaling is involved in different metastatic cancers, and its role in chemoresistance is well documented. Thus, inhibition of EGFR activation and pAKT as a complement to TKI-based therapy can reverse cellular mechanisms, leading to an attenuation of chemoresistance. A previous study on the alterations of EMT markers in lung cancer demonstrated that decreased E-cadherin or increased fibronectin levels are associated with poor survival outcomes.[47] Inhibition of tumor invasion or migration is one of the goals of suppressing CSC properties, especially in patients who have metastases instead of only a primary lesion. The results of Boyden chamber assays showed the enhancement of the long-term effects of combined NNK and arecoline exposure on SCC25 and FaDu cell motility and migration, which are integral steps in the metastatic cascade of tumor cells, suggesting that long-term exposure to NNK and arecoline can promote invasion and metastasis to distal sites.

As described above, mounting evidence also suggests a link between CSCs and the EMT. Herein, we showed that long-term exposure to NNK and arecoline in HNSCC cells induced the development in CSCs linked with the EMT, which is key for tumor progression. From the standpoint of CSC-related gene products, validated stemness gene-related proteins, such as Nanog and OCT4, support the invasive competence of tumor cells, which can self-renew.[48–50] Based on our data, overexpression of ALDH1, Nanog, and OCT4 was also found in long-term NNK- and arecoline-treated cells compared with the levels in control HNSCC cells. In this study, as in other reports, no specific marker of CSC properties in HNSCC was delineated, and the relevance of other CSC-associated markers, such as CD24/CD44^+^ or CD133, is still debated.[51, 52]

**Fig 6:**
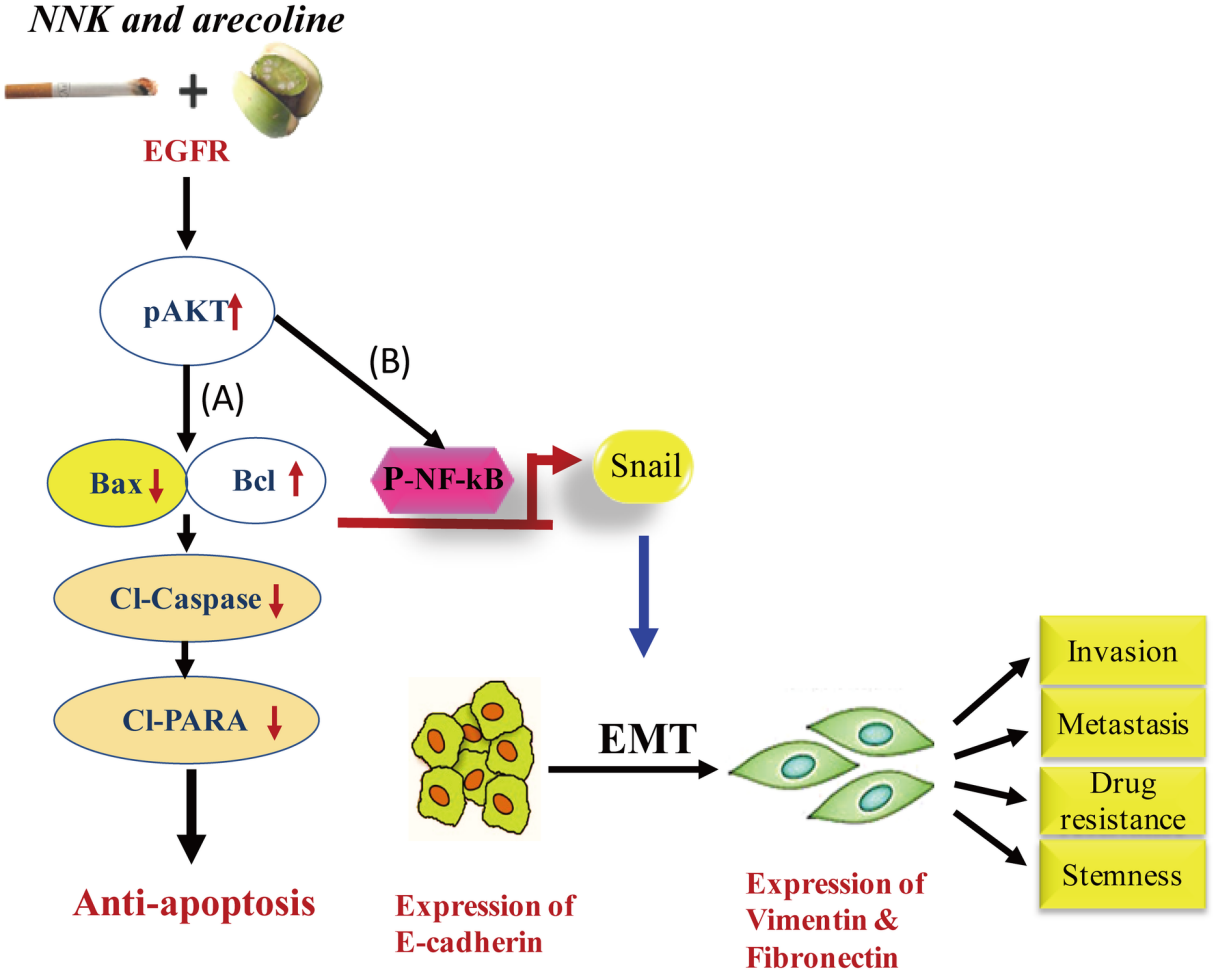
Proposed model to illustrate NNK- and arecoline-mediated EGFR/AKT/NFκB signal activation resulting in enhanced anti-apoptosis, EMT, and tumor progression in HNSCC cells. **A**. Long-term exposure to NNK and arecoline synergistically increased anti-apoptotic activity via the EGFR/AKT pathway. **B**. Such exposure also synchronized the induction of the EMT phenomenon with morphological alterations via NFκB/Snail signaling, ultimately leading to tumor progression, drug resistance, and stemness.

In summary, we demonstrated that long-term exposure to NNK combined with arecoline activated EGFR/AKT signaling and was involved in anti-apoptosis, CSC properties, and Cis resistance in HNSCC cells. These findings provide new insights into the potential molecular mechanisms of long-term NNK- and arecoline-induced anti-apoptosis and CSC characteristics in head and neck cancer cells. The results presented here suggest that both NNK and arecoline not only are strong carcinogens by themselves but also can synergistically promote the growth and progression of HNSCC cells when administered in combination. The ability of NNK and arecoline to synergistically promote proliferation, adherence-independent growth, EMT, and CSC properties, which could be inhibited by administering an anti-EGFR antibody, suggest that NNK and arecoline might contribute significantly to the growth and metastasis of EGFR-sensitive tumors. Targeting this pivotal receptor when managing tumor aggressiveness and therapeutic resistance might prove beneficial in treating HNSCC, as shown in our proposed model (Fig 6). Our data suggest that understanding these mechanisms is critical in developing effective therapeutic strategies with the potential of CSC inhibition in treating tumors that develop after NNK and arecoline exposure.
